# Trait selection and co-existence of phytoplankton in partially mixed systems: Trait based modelling and potential of an aggregated approach

**DOI:** 10.1371/journal.pone.0194076

**Published:** 2018-03-22

**Authors:** Frank Peeters, Dietmar Straile

**Affiliations:** Limnological Institute, University of Konstanz, Konstanz, Germany; National Taiwan University, TAIWAN

## Abstract

Trait selection and co-existence in phytoplankton communities in partially mixed water columns is investigated using trait based modelling. In the models employed, trait selection results from phytoplankton competition for two limiting resources, light and nutrients. The study employs spatially resolved models, in which the phytoplankton community is represented as a large number of trait-groups characterized by fixed trait combinations (trade-offs). Results from the trait-group resolving model (RM) are compared to results from an aggregated trait based model with adaptive traits (AM). Differences in specific production resulting from a trade-off between the half saturation constants of light and nutrients are sufficient to support evolutionary stable co-existence confirming that co-existence does not require differences in resource consumption. If abiotic conditions lead to the selection of a single trait group in RM, AM provides excellent approximations of the development of total biomass, average community trait and trait variance in the phytoplankton community. However, if selection leads to bimodal trait distributions, e.g. to co-existence of two trait groups (or species), functionally important properties of the phytoplankton community cannot be adequately represented by the aggregated information provided by AM. Because the increase in variance due to the development of bimodal trait distributions cannot be distinguished from an increase in variance due to an increase in trait diversity, the development of trait variance in AM models is not a reliable measure of trait diversity. Furthermore, AM may not provide reliable simulations of trophic interactions if the performance of the consumers depends on the traits of their resources. However, AM may support exploration of the consequences of environmental conditions and of the parameterization of species for co-existence within communities.

## Introduction

The functioning and performance of aquatic ecosystems depend on the structure of the aquatic community. Composition and dynamics of the community result from the response of the individual species and their interactions to changes in environmental conditions and in the structure of the community itself. The study here focusses on phytoplankton communities in partially mixed water columns and investigates the consequences of competition for light and nutrients on community dynamics and composition, and specifically on selection and co-existence of phytoplankton.

In general, the prediction of community properties and dynamics based on the development of individual species is difficult because detailed information is required to describe the performance of the individual species and the often non-linear interactions between them. As the functional traits of a species determines its fitness in a given environment, functional traits have been rediscovered in recent years as promising basis for the prediction of community development [1]. The seasonal dynamics and community structure of phytoplankton in response to limiting resources can be well-predicted from lab-determined functional traits [2,3] suggesting “that functional traits provide a mechanistic foundation for community ecology, and that variation in community structure is predictable in spite of the complexity of ecological communities” [2].

Long-term co-existence in homogeneous systems requires that species differ in their resource consumption [4]. Consistently, stable co-existence of phytoplankton competing for light and nutrients in vertically fully mixed systems requires that co-existing species differ in traits related to resource consumption, i.e. in their specific light extinction coefficient *k* divided by the product of their nutrient to carbon ratio *γ* and specific loss rate *l*_*bg*_, (*k* / *(γ·l*_*bg*_) [5,6]. In partially mixed systems the condition for co-existence of phytoplankton is more complicated than in fully mixed systems: Ryabov et al. [7] considered competition between two neutrally buoyant species which co-existed if the species differed in traits characterizing resource consumption, i.e. in their *k*/*γ* ratio. However, they showed that in the case of a sub-surface layer of phytoplankton or a deep chlorophyll maximum (DCM) differences in half saturation constants can modify the effects of differences in consumption rates [7]. Recently, a simulation with an eco-evolutionary model that considered adaptation of monomorphic phytoplankton species with respect to half saturation constants for light and nutrients in partially mixed systems revealed co-existence of three species each corresponding to a single point in trait space [8]. Although this study did show co-existence in the absence of consumption differences, it did not discuss the role of consumption differences versus production differences, i.e. differences in half-saturation constants, for allowing co-existence of phytoplankton species.

Here we investigate the dynamics of phytoplankton communities in partially mixed systems. The phytoplankton communities are composed of species differing only in traits related to specific production but not with respect to resource consumption. We study the influence of nutrient enrichment and mixing intensity on the occurrence of long-term co-existence. We further investigate whether the selected community is restricted to very few narrow trait groups as assumed in the genetic selection process used by [8].

Predictions of the response of aquatic ecosystems to changes in environmental conditions are usually based on mathematical models that are typically solved numerically. Models have always been concerned with the traits of species, as mathematically the model parameters describing the performance of species correspond to the values of the traits of the species. However, representation of plankton communities based on the traits of the numerous individual species making up the community is usually not a practicable approach because model performance could become slow and many of the parameters required to represent the traits of the different species are not available or uncertain. Increasing the number of species considered in a model may therefore not necessarily increase predictive abilities of the model [9,10].

Instead of considering a large number of species with different combinations of traits, trait based modelling represents communities by functional traits assuming that the trait space is continuous and trade-offs between traits can be described by functional relationships. The dynamics of the traits of a community can be simulated by resolving the continuous trait space using a finite number of discrete trait groups. However, this approach requires the dynamic simulation of a large number of such trait groups and is therefore computationally not more efficient than species based modelling of community dynamics.

Recently, aggregated trait-based modeling using adaptive traits to dynamically simulate average community properties has been suggested as an alternative approach to investigate community dynamics (e.g. [11–16]). Aggregated trait based modelling with adaptive traits does not resolve details of the communities investigated but only models few statistical properties of the community, typically the community biomass and the average and the variance of the community trait distribution. The models consider adaptive traits as they dynamically simulate the change of the average community trait in response to changes in environmental conditions and community properties. According to [17] trait variance is a key property in trait-based analysis as it is a measure of trait diversity and it is proportional to the rate at which the average community trait changes. Hence, aggregated trait based modeling with adaptive traits requires the simulation of the temporal changes in average community traits and of the trait variance.

Resolved and aggregated trait based models have both limitations. Whereas resolved models discretize trait space to a limited number of trait groups and therefore only approximate the development of communities consisting of species occupying continuous trait space, aggregated models consider continuous trait space but can only simulate aggregated statistical properties of the trait distributions. Comparison between results from aggregated trait based models using adaptive trait simulation and simulations with resolved models are scarce and typically have focused on predator prey interactions in zero dimensional systems (e.g. [12,18]). Comparisons of aggregated trait based models with field observations are rare and typically limited to models that assume fully mixed conditions (e.g. [15,16]). An exception is [19] comparing results from a vertically resolved aggregated trait based model with oceanic observations. Whether aggregated trait based models considering competition of phytoplankton in vertically resolved aquatic systems can be employed to assess the consequences of vertical transport on plankton community dynamics and co-existence has not yet been explored in detail.

The dynamics of phytoplankton communities in lakes and oceans are strongly affected by transport due to vertical turbulent diffusion and sinking, because the position of phytoplankton within the water column determines availability of light and nutrients for production. The persistence and dynamics of single phytoplankton species in vertical water columns have been demonstrated to strongly depend on vertical turbulent diffusion (e.g. [20–23]). Intensity of vertical mixing also affects the outcome of competition between phytoplankton species in vertically resolved systems (e.g. [7,24–28]).

Most modelling studies investigating phytoplankton competition have considered the simultaneous interaction of very few explicitly modelled species with prescribed trait combinations and e.g. have tested whether these species can co-exist. Typically, the species considered in these models differ in several parameters describing specific production and resource consumption. Very few studies have considered a large number of phytoplankton species in vertically resolved systems to investigate the development of community structure, e.g. [29]. The latter study suggested the use of aggregated trait based approaches to reduce computational effort. Because vertical transport strongly affects competition of light and nutrient limited phytoplankton such aggregated trait based models need to include transport processes to be able to adequately simulate phytoplankton communities in lakes and oceans.

The study here employs vertically resolved models and uses two different approaches to simulate the community dynamics of phytoplankton: In the first approach the temporal changes in the phytoplankton community are simulated by resolving a large number of fixed trait combinations each representing a different trait-group, which can also be considered a species. In the second approach the development of statistical properties of the community trait distributions is simulated using aggregated trait based models with adaptive traits.

In all our simulations phytoplankton competes for two limiting resources, light and nutrients, which both vary with depth and depend on the phytoplankton development in the water column. The phytoplankton species are assumed to differ not with respect to resource consumption, but only with respect to specific production, i.e. the species differ only in their half saturation constant for light, *H*_*I*_, and their half saturation constant for nutrients, *H*_*N*_. The half saturation constants *H*_*I*_ and *H*_*N*_ will affect the degree of light and nutrient limitation, and are linked by a trade-off function. Hence, the differences between all phytoplankton species can be described by *H*_*I*_ alone and *H*_I_ may therefore be considered a master trait characterizing the differences within the phytoplankton community.

In trait based approaches trait-space is typically assumed to be continuous, i.e. the species in the phytoplankton community can assume any value of the half saturation constant *H*_*I*_ within an interval of trait values bounded by the evolutionary possible minimum and maximum *H*_*I*_ of the community. The development of long term stable co-existence in a phytoplankton community consisting of numerous species that differ in master trait *H*_*I*_ can be interpreted as the evolutionary stable co-existence for the specific trade-off between the half saturation constants *H*_*I*_ and *H*_*N*_.

Utilizing numerical experiments we address the following hypotheses:

1. In models resolving a large number of individual species and also in aggregated trait-based models differences in specific production of phytoplankton, here resulting from differences in the half saturation constants *H*_*I*_ and *H*_*N*_, can be sufficient to support co-existence in partially mixed water columns. In both models, differences in parameters related to resource consumption, e.g. to light extinction or stoichiometry, are not necessarily required for co-existence.
2. Evolutionary stable co-existence of phytoplankton differing only in traits related to specific production is possible at low but not at high mixing intensities.
3. The statistical properties of trait distributions derived from trait based modelling of a large number of individual trait groups agree with those simulated with aggregated adaptive trait based models.
4. Aggregated adaptive trait based modelling can be employed to assess the conditions under which evolutionary stable co-existence occurs.

## Methods

### The model and analysis of model results

The model applied in this study considers a community of light and nutrient limited phytoplankton in a partially mixed vertical water column. Individual phytoplankton species are simulated as in e.g. [24]. The model dynamically simulates the concentration of carbon *C*_*i*_ of the phytoplankton species *i*, nutrient concentration in the water column, *N*, and a nutrient pool in the sediments *N*_*S*_:

$$\frac{\partial C_{i}\left(t,z\right)}{\partial t}=p_{spec,i}\left(I,N\right)\cdot C_{i}-l_{bg,i}\cdot C_{i}-v_{i}\cdot \frac{\partial C_{i}}{\partial z}+\frac{\partial }{\partial z}\left(K_{z}\cdot \frac{\partial C_{i}}{\partial z}\right)$$ (1)

$$\begin{array}{l} \frac{\partial N\left(t,z\right)}{\partial t}=-\sum \gamma_{i}\cdot \left(p_{spec,i}\left(I,N\right)\cdot C_{i}-l_{bg,i}\cdot C_{i}\;\right)\;\;+\frac{\partial }{\partial z}\left(K_{z}\cdot \frac{\partial N}{\partial z}\right)\\ \frac{dN_{s}\left(t\right)}{dt}=\sum v_{i}\cdot \gamma_{i}\cdot C_{i}\left(z_{\text{max}}\right)-m\cdot N_{s}\\ p_{spec,i}\left(I(z),N(z)\right)=\mu_{\text{max},i}\frac{I(z)}{H_{I,}{}_{i}+I(z)}\cdot \frac{N(z)}{H_{N}{}_{,i}+N(z)}\\ I\left(z\right)=I_{0}\text{exp}\left(-\int_{0}^{z}\sum \left(k_{i}\cdot C_{i}+k_{bg}\right)\cdot dz\right) \end{array}$$

Thereby, *t* represents time, *z* is depth below the water surface and *z*_*max*_ is *z* at maximum water column depth. Specific production *p*_*spec*,*i*_ of species *i* is limited by light intensity *I* and nutrient concentration *N*. *μ*_*max*,*i*_ is the maximum specific algal production rate, *H*_*I*,*i*_ the half-saturation constant of light-dependent algal production and *H*_*N*,*i*_ the half saturation constant with respect to nutrients of species *i*. The limitation of production by light and nutrients is simulated using a product law. Note that Liebig’s minimum law is not compatible with aggregated adaptive trait based models if phytoplankton differs in trait *H*_*I*_ because the solution of these models require the derivative of specific net production with respect to the traits (see Eq 5). The vertical profile of light intensity is calculated by considering not only background attenuation but also light attenuation by algae which leads to integro-partial-differential equations [20]. *k*_*i*_ is the specific light-attenuation coefficient of algal biomass of species *i*, *k*_*bg*_ the background light attenuation. Algae and nutrients are transported by turbulent mixing which is modelled as a diffusive type process that is characterized by a vertical turbulent diffusivity *K*_*z*._ Sedimentation of algae is simulated using a sinking velocity *v*_*i*_. As bottom boundary condition for the dissolved nutrients the model considers the nutrient flux from a sediment pool of nutrients (*N*_*S*_) as in [22,23]. The sediment nutrient pool is filled with nutrients stored in the phytoplankton sedimenting out of the water column into the sediments. These nutrients are re-mineralized at rate *m* and returned to the bottom of the water column (see [23]). The fluxes of phytoplankton and dissolved nutrient concentrations at the surface (*F*_*s*_) and the bottom (*F*_*b*_) of the water column are *F*_*sCi*_ = 0; *F*_*bCi*_ = *v*_*i*_*·C*_*i*_(z_max_); *F*_*sN*_ = 0; *F*_*bN*_ = −*m·N*_*s*_. All phytoplankton species are assumed to have the same specific maximum growth rate *μ*_*max*_, specific loss rate *l*_*bg*_, specific light extinction coefficient *k*, nutrient to carbon ratio *γ*, and sedimentation velocity *v*, but to differ in their half saturation constants for light *H*_*I*_ and nutrients *H*_*N*_. The trade-off between *H*_*I*_ and *H*_*N*_ is modelled using a power law:

$$\text{ln}\left(H_{N}\right)=a+b\cdot \text{ln}\left(H_{I}\right)$$ (2)

Thus, the phytoplankton species in the model can be considered as trait groups characterized by a master trait *H*_*I*_. The phytoplankton equation can be condensed into the form:

$$\frac{dC_{i}\left(t,z\right)}{dt}=r\left(H_{I}{}_{i},I,N\right)\cdot C_{i}-v\cdot \frac{\partial C_{i}}{\partial z}+\frac{\partial }{\partial z}\left(K_{z}\cdot \frac{\partial C_{i}}{\partial z}\right),$$ (3)

and *r* is the specific net growth rate *r* = *p*_*spec*_(*H*_*Ii*_,*I*,*N*) − *l*_*bg*_.

The development of the phytoplankton community is simulated using two different approaches:

1. Discrete trait groups with different values of the master trait *H*_*I*_ are resolved and simulated simultaneously (resolved model RM). Specifically, we consider 101 trait groups that cover *H*_*I*_ between 20 and 120 μmol photons m^-2^ s^-1^ in steps of 1 μmol photons m^-2^ s^-1^.
2. Statistical properties of the community trait distribution *c*(*s*,*z*), i.e. the concentration per unit trait at trait *s* at depth *z*, are simulated using an aggregated adaptive trait based community model (AM). As continuous master trait *s* the model uses half saturation constant of light *H*_*I*_. The model AM considers as state variables total phytoplankton concentration of the community *C*_*T*_ = ∫ *c* (*H*_*I*_) · *dH*_*I*_ and first and second moment of the community trait distribution *M*_1_ = ∫ *c*(*H*_*I*_) · *H*_*I*_ · *dH*_*I*_ and, $M_{1}=\int c(H_{I})\cdot H_{I}^{2}\cdot dH_{I}$ respectively, whereby the integration is over trait space. The total community concentrations and the first moment of the trait distribution provide the average community trait *Tr*_*av*,*M*_ = *M*_1_ / *C*_*T*_, and the second moment *M*_*11*_ the variance of the trait distribution around the average community trait $Tr_{\text{var},M}=M_{11}/C_{T}-Tr_{av,M}^{2}$.

The temporal changes of *C*_*T*_, *M*_*1*_ and *M*_*11*_ are given by:

$$\begin{array}{l} \frac{\partial C_{T}\left(t,z\right)}{\partial t}=\int r\left(H_{I},I,N\right)\cdot c\left(H_{I}\right)\cdot dH_{I}-v\frac{\partial C_{T}}{\partial z}+\frac{\partial }{\partial z}\left(K_{z}\frac{\partial C_{T}}{\partial z}\;\right)\\ \frac{\partial M_{1}\left(t,z\right)}{\partial t}=\int r\left(H_{I},I,N\right)\cdot c\left(H_{I}\right)\cdot H_{I}\cdot dH_{I}-v\frac{\partial M_{1}}{\partial z}+\frac{\partial }{\partial z}\left(K_{z}\frac{\partial M_{1}}{\partial z}\;\right)\;\\ \frac{\partial M_{11}\left(t,z\right)}{\partial t}=\int r\left(H_{I}I,N\right)\cdot c\left(H_{I}\right)\cdot H_{I}{}^{2}\cdot dH_{I}-v\frac{\partial M_{11}}{\partial z}+\frac{\partial }{\partial z}\left(K_{z}\frac{\partial M_{11}}{\partial z}\;\right)\; \end{array}$$ (4)

whereby the state variables, light intensity, nutrient concentration and the community trait distribution *c* are all dependent on *z*. The integrals in these differential equations are approximated using a Tailor expansion around the average community trait *M*_1_/*C*_*T*_. The Taylor expansion leads to a dependence of the first and second moment on the 3^rd^ and 4^th^ moment of the trait distribution. As the temporal development of moments of the trait distribution always depends on higher order moments a closure scheme is required for the solution of the aggregated trait based model.

Adopting the approach of [16] and [12], the integrals in Eq 4 were approximated using a Taylor expansion and the assumption that the community trait distributions are of Gaussian form:

$$\begin{array}{l} \int r\left(H_{I},I,N\right)\cdot c\left(H_{I}\right)\cdot dH_{I}\;\approx \\ \;\;\;\;\;\;\;\;\;\;\;\;\;\;\;\;\;\;\;\;\;\;\;\;\;\;\;\;\;\;\;r_{m}\cdot C_{T}+\frac{1}{2}\cdot \left(M_{11}-\frac{M_{1}{}^{2}}{C_{T}}\right)\;\;\;\cdot {\frac{\partial^{2}r}{\partial H_{I}^{2}}|}_{M_{1}/C_{T}}\\ \int r\left(H_{I},I,N\right)\cdot c\left(H_{I}\right)\cdot H_{I}\cdot dH_{I}\;\approx \\ \;\;\;\;\;\;\;\;\;\;\;\;\;\;\;\;\;\;\;\;\;\;\;\;\;\;\;\;\;\;r_{m}\cdot M_{1}+\left(M_{11}-\frac{M_{1}{}^{2}}{C_{T}}\right)\cdot {\frac{\partial r}{\partial H_{I}}|}_{M_{1}/C_{T}}+\frac{1}{2}\cdot \left(\frac{M_{1}\cdot M_{11}}{C_{T}}-\frac{M_{1}{}^{2}}{C_{T}{}^{2}}\right)\;\cdot {\frac{\partial^{2}r}{\partial H_{I}^{2}}|}_{M_{1}/C_{T}}\\ \int r\left(H_{I},I,N\right)\cdot c\left(H_{I}\right)\cdot H_{I}^{2}\cdot dH_{I}\approx \\ \;\;\;\;\;\;\;\;\;\;\;\;\;\;\;\;\;\;\;\;\;\;\;\;\;\;\;\;\;\;\;r_{m}\cdot M_{11}+2\cdot \left(\frac{M_{1}\cdot M_{11}}{C_{T}}-\frac{M_{1}{}^{3}}{C_{T}{}^{2}}\right)\cdot {\frac{\partial r}{\partial H_{I}}|}_{M_{1}/C_{T}}+\frac{1}{2}\cdot \left(2\frac{M_{1}{}^{4}}{C_{T}{}^{3}}-5\frac{M_{1}{}^{2}\cdot M_{11}}{C_{T}{}^{2}}+3\frac{M_{11}{}^{2}}{C_{T}}\right)\cdot {\frac{\partial^{2}r}{\partial H_{I}^{2}}|}_{M_{1}/C_{T}} \end{array}$$ (5)

with *r*_*m*_ = *r* (*M*_1_/*C*_*T*_,*I*,*N*) defined as the specific net growth rate at the average community master trait *H*_*I*_. The trait *H*_*N*_ is calculated from *H*_*I*_ using the trade-off function given in Eq 2. Because the values of all other traits are assumed to be the same for all phytoplankton species, the equations for dissolved nutrients, the nutrient concentration in the sediment, and the vertical profile of light intensity are given by:

$$\begin{array}{l} \;\frac{\partial N\left(t,z\right)}{\partial t}=-\gamma \cdot \;\left(r_{m}\cdot C_{T}+\frac{1}{2}\cdot \left(M_{11}-\frac{M_{1}{}^{2}}{C_{T}}\right)\;\;\;\cdot {\frac{\partial^{2}r}{\partial s^{2}}|}_{M_{1}/C_{T}}\right)+\frac{\partial }{\partial z}\left(K_{z}\frac{\partial N}{\partial z}\;\right)\\ \frac{\partial N_{s}\left(t,z\right)}{\partial t}=v\cdot \gamma \cdot C_{T}\left(z_{\text{max}}\right)-m\cdot N_{s}\\ I\left(t,z\right)=I_{0}\text{exp}\left(-\int_{0}^{z}\left(k\cdot C_{T}\left(t,z\right)+k_{bg}\right)dz\right) \end{array}$$ (6)

The full set of differential equations and the specific assumptions of the AM are provided in Appendix A in S1 File. Variables and parameters employed in the simulations are summarized in Table A in S1 File. The trade-off function between *H*_*I*_ and *H*_*N*_ is depicted in Figure A in S1 File.

The final equations do not provide exact solutions to Eq 4 because of the Taylor approximation and the assumption of normally distributed traits. However, Eqs 5 and 6 provide approximations to the correct solution with the similar limitations as in earlier studies using aggregated trait based models [12,15,16].

The same initial condition for the trait distribution of the phytoplankton community was used in AM and RM simulations, i.e. in both model types we used at all water depths a normal distribution with a total phytoplankton concentration *C*_*T*_ = 200 mgC m^-3^, a mean trait *H*_*I*_ = 70 μmol photons m^-2^ s^-1^ and a community trait variance of 100 (μmol photons m^-2^ s^-1^)^2^ around the mean trait as initial condition. The initial nutrient concentration in the sediment was *N*_*Sini*_ = 0 mgCm^-2^. We analysed competition with a range of initial nutrient concentrations *N*_*ini*_ between 10 and 90 mgP m^-3^ and a range of turbulent diffusivities between 2 and 1 x 10^−3^ m^2^ s^-1^. Incident light intensity was *I*_*0*_ = 300 μmol Photons m^-2^ s^-1^ and the maximum water depth was *z*_*max*_ = 50 m.

The model equations were solved numerically by discretizing the spatial dimension in 0.5 m steps. The advection equation was discretized using a third order upwind scheme as in [20,24]. The remaining system was solved as in [22] using the ode15s solver of MATLAB for the resulting system of coupled ordinary differential equations.

### Model comparison and statistical properties

The properties *C*_*T*_, *M*_1_ and *M*_11_ simulated with AM include the entire continuous trait space whereas in RM the trait space is limited by the range of the trait groups considered, i.e. RM considers 101 trait groups located at *H*_*I*_ from 20 to 120 μmol Photons m^-2^ s^-1^ in steps of 1 μmol Photons m^-2^ s^-1^. Thus, for the comparison of the model results obtained with AM and RM we derived from the statistical properties provided by AM concentrations of the trait groups *C*_*i*_ at the same trait values as in RM. The concentration density distribution *c*(*H*_*I*_) was calculated from *C*_*T*_, *M*_1_ and *M*_11_ simulated with AM assuming a Gaussian distribution. The concentrations *C*_*i*_ of AM for the same trait groups as in RM were obtained by integrating *c*(*H*_*I*_) over intervals of 1 μmol Photons m^-2^ s^-1^ centered at the *H*_*Ii*_ considered in RM. These concentrations *C*_*i*,_ were utilized to calculate all statistical properties of the trait distribution and diversity indices mentioned below. Based on the *C*_*i*_ obtained from AM and RM total concentration *C*_*T*_, average trait *Tr*_*av*_, and trait variance *Tr*_*var*_ of the phytoplankton community within the trait range of RM were calculated from:

$$\begin{array}{l} C_{T}=\sum_{i}C_{i};\;\;\;M_{1}=\sum_{i}C_{i}\cdot H_{I,i};\;\;\;M_{11}=\sum_{i}C_{i}\cdot H_{I,i}^{2}\\ Tr_{av}=\frac{M_{1}}{C_{T}}\\ Tr_{\text{var}}=\frac{M_{11}}{C_{T}}-Tr_{av}^{\;\;2} \end{array}$$ (7)

All diversity indices and properties characterizing the trait distributions were calculated from the *C*_*i*_ obtained with RM and the *C*_*i*_ derived from AM. Functional richness, *F*_*rich*_, is the number of trait groups with concentrations larger than 10^−3^ mgC m^-3^. A threshold is required for richness calculations because in numerical simulations disappearing species do not become extinct, but only asymptotically approach zero concentrations. Our results are robust regarding the magnitude of the threshold, e.g. similar conclusions are obtained for a threshold of 10^−6^ mgCm^-3^ or whether it was defined as 0.01% of the maximum concentration.

The index of functional divergence (*FD*_*80*_) employed in this study is a generalized form of the functional divergence index described in [30] and quantifies a range in trait space that is occupied by 80% of the total phytoplankton concentration utilizing the 10% and 90% percentile in the cumulated trait distribution. The trait range obtained is normalized by the maximum range of trait space:

$$FD_{80}=\frac{H_{I,90}-H_{I,}{}_{10}}{H_{I,\text{max}}-H_{I,0}},\;\;\text{with}\;\;\int_{H_{I,0}}^{H_{I,10}}c\left(H_{I}\right)dH_{I}=0.1\cdot C_{T}\;\;\;\text{and}\;\;\int_{H_{I,0}}^{H_{I,90}}c\left(H_{I}\right)dH_{I}=0.9\cdot C_{T}$$ (8)

and *H*_*I*,*0*_ is the smallest and *H*_*I*,*max*_ the largest trait value *H*_*I*_ considered, i.e. 20 and 120 μmol m^-2^ s^-1^, respectively. The integrals in Eq 8 implicitly provide *H*_*I*,*10*_ and *H*_*I*,*90*_, which are the trait values at the limits of the central trait region occupied by 80% of the phytoplankton community. Since we compare discrete trait groups with concentrations *C*_*i*_ the integrals in Eq 8 were replaced by sums of *C*_*i*_ obtaining the boundaries of the occupied trait range by summing starting from the lowest *H*_*Ii*_ until the threshold of 0.1·*C*_*T*_ and 0.9·*C*_*T*_, respectively, is exceeded for the first time.

In addition to the functional diversity indices we determined the trait value *Tr*_*Cmax*_ at maximum concentration *C*_*max*_. *C*_*max*_ is the maximal concentration in the trait distribution *C*_*i*_ obtained from RM or determined from the simulation results of AM. Further, we calculated the ratio between the concentration at average community trait *C*_*Trav*_ and *C*_*max*_:

$$f_{CTrav/Cmax}=\frac{C_{Trav}}{C_{\text{max}}}$$ (9)

## Results

**Fig 1:**
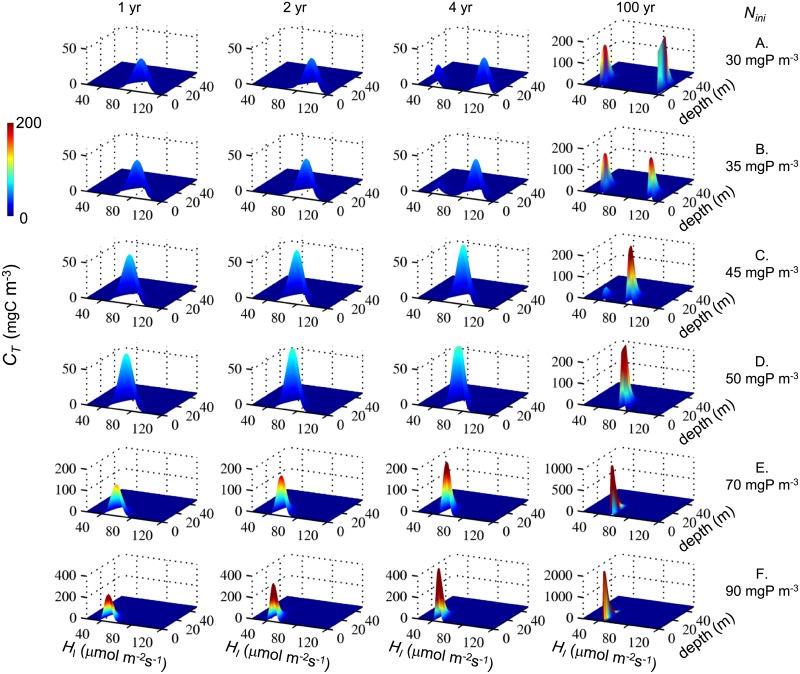
Temporal development of trait distributions at different nutrient enrichments (*N*_*ini*_) (A-F) simulated with RM. Displayed are concentration (mgC m^-3^) in 101 equally spaced trait intervals as function of master trait *H*_*I*_ (μmol Photons m^2^ s^-1^) and depth (m). In all simulations turbulent diffusion is *K*_*z*_ = 5·10^−5^ m^2^ s^-1^.

**Fig 2:**
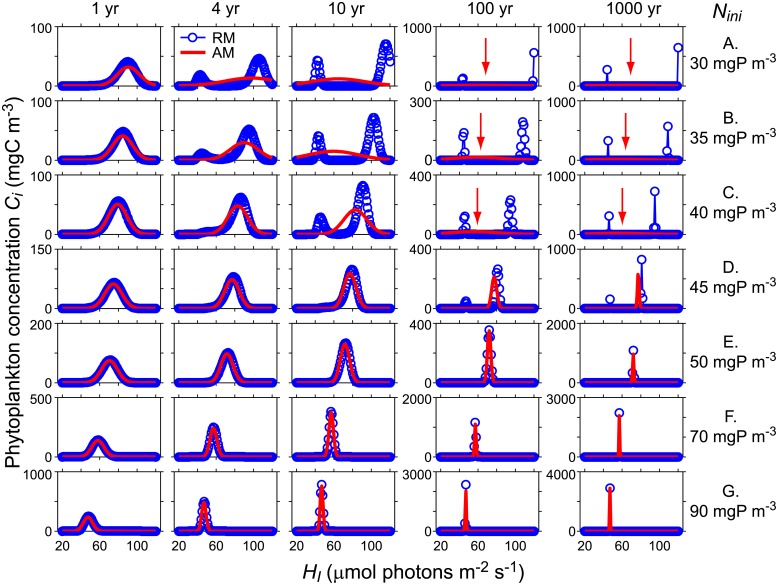
Comparison of the temporal development of trait distributions simulated with RM (blue) and AM (red) with *N*_*ini*_ ranging from 30 to 90 mgP m^-3^ (Fig A-G). Displayed are trait distributions at 0.75 m water depth (depth of the center of the second grid cell from the surface) resolving 101 discrete equally spaced trait intervals. The red arrows indicate the location of *Tr*_*av*_ in AM for simulations in which *Tr*_*var*_ is very large. In all simulations turbulent diffusion was *K*_*z*_ = 5·10^−5^ m^2^ s^-1^.

**Fig 3:**
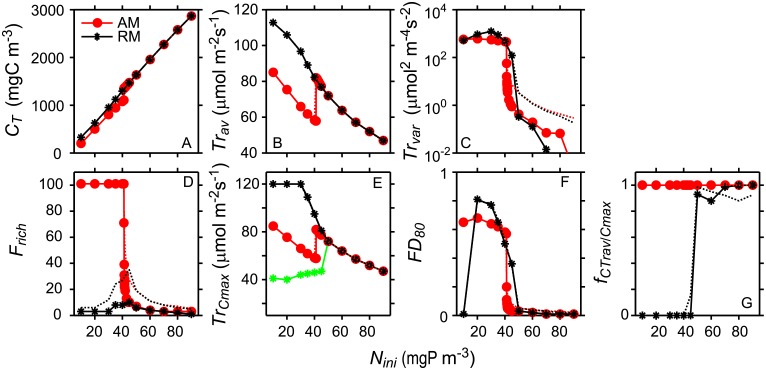
Vertical distribution of aggregated properties of the trait distributions simulated with RM and AM at two *N*_*ini*_ resulting in either unimodal or bimodal trait distributions (*N*_*ini*_ = 35 mgP m^-3^, bimodal, Fig A-C; *N*_*ini*_ = 50 mgP m^-3^, unimodal, Fig E-G). The trait distributions at 0.75m and 9.75 m depth predicted by RM and AM are shown in (D) for *N*_*ini*_ = 35 mgP m^-3^ and in (H) for *N*_*ini*_ = 50 mgP m^-3^. The aggregated properties obtained from RM and AM are indicated by black and red lines respectively. Depicted are total concentration *C*_*T*_ (A, E), average trait *Tr*_*av*_ and trait at maximum concentration *Tr*_*cmax*_ (B,F) and trait diversity *FD*_*80*_ (C,G). Note that *Tr*_*cmax*_ ofRM is indicated by a green line whereas in AM *Tr*_*cmax*_ = *Tr*_*av*_ and therefore identical to the red line. In all simulations turbulent diffusion was *K*_*z*_ = 5·10^−5^ m^2^ s^-1^ and simulation time was 1000 yr.

The simulations with RM indicate that competition of phytoplankton for light and nutrients results in a strong selection of species. At a vertical diffusivity of 5·10^−5^ m^2^s^-1^ the selection of traits, i.e. survival of trait groups with specific traits, and the co-existence of trait groups depend on the nutrients initially available in the system (Fig 1). For initial nutrient concentrations *N*_*ini*_ < 50 mgP m^-3^ the trait distribution of the community develops within a 100 yr time period from an initial unimodal normal trait distribution into a bimodal trait distribution (Fig 1A–1C). The bimodal trait distributions after 100yr and 1000 yr simulation time are characterized by high abundances in two very narrow trait regions that are separated in trait space indicating extinction of trait groups with intermediate *H*_*I*_ (Figs 1A–1C and 2A–2D). At *N*_*ini*_ ≤ 30 mgP m^-3^ one of the co-existing trait groups has a *H*_*I*_ at the upper boundary of the trait space considered to be available for the community (see Figs 1A and 2A for *N*_*ini*_ = 30 mgP m^-3^). With nutrient enrichment, the value of *H*_*I*_ of the co-existing trait group is shifted away from the upper boundary of the trait space. The larger *N*_*ini*_ the smaller is the difference between the *H*_*I*_ at the two local maxima of the final bimodal trait distribution. At *N*_*ini*_ ≥ 50 mgP m^-3^ the trait distribution does not develop into a bimodal distribution but remains unimodal with essentially one species out-competing all other species after 100 yrs and 1000 yrs development time (Figs 1D–1F and 2E–2G). The larger *N*_*ini*_ the lower is *H*_*I*_ of the surviving species. The traits selected by competition are independent of the depth within the water column (Figs 1 and 3).

**Fig 4:**
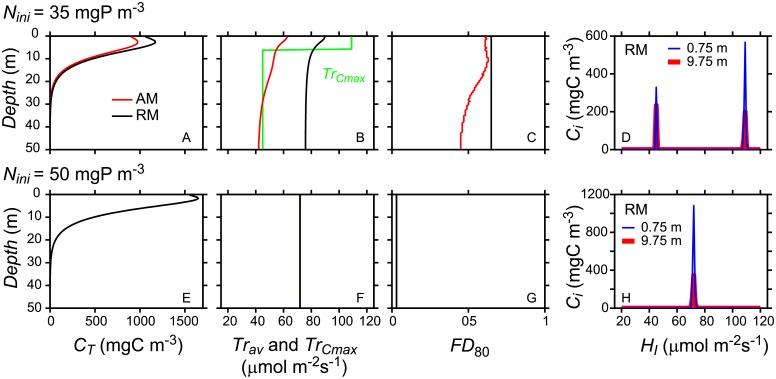
Aggregated properties of the trait distributions, diversity indices and additional characteristics of the trait distributions as a function of nutrient enrichment (*N*_*ini*_) after 100 years (dotted lines) and 1000 years (symbols and solid lines) simulation time. Results are based on simulations with RM (black) and AM (red). (A) Total concentration (*C*_*T*_), (B) Community average of the master trait *H*_*I*_ (*TR*_*av*_), (C) Community trait variance (*TR*_*var*_) of master trait *H*_*I*_, (D) Functional richness (*F*_*rich*_), i.e the number of trait groups exceeding 10^−3^ mgC m^-3^, (E) master trait *H*_*I*_ at maximum concentration *C*_*max*_ (*Tr*_*Cmax*_), (F) Functional divergence (*FD*_*80*_), and (G) ratio between concentration at *Tr*_*av*_ and the maximum concentration (*f*_*CTrav/Cmax*_). All characteristics shown were recorded at 0.75 m water depth. The symbols indicate *N*_*ini*_ for which simulations were performed, the lines connect these results, but do not imply that simulation results must fall on these lines. I.e. it is not clear whether AM predicts a smooth transition or a discontinuous jump from low to high values *Tr*_*av*_ around 40 mgP L^-1^. In all simulations turbulent diffusion was *K*_*z*_ = 5·10^−5^ m^2^ s^-1^.

Model results for the trait distribution obtained with AM agree very well with the results from RM as long as the trait distributions are unimodal, i.e. at *N*_*ini*_ ≥ 50 mgP m^-3^ (Figs 2 and 4). In these cases total concentration *C*_*T*_, average community trait and variance of the trait distribution, functional richness, index of functional divergence, as well as trait at maximum concentration and concentration ratio at maximum trait agree very well between RM and AM (Fig 4). In cases in which RM simulated bimodal trait distributions (i.e. at *N*_*ini*_ < 50 mgP m^-3^), AM predicts average community traits that are typically between the trait values at the two local maxima of the bimodal distribution predicted by RM (Fig 2A–2D red arrows, and Fig 4E). The variance of the trait distribution simulated with AM is large, implying very broad trait distributions, and similar to the trait variance determined from the results of RM (Fig 4C). The total concentration of the phytoplankton community after 100 and 1000 years of development agree well between RM and AM independent of *N*_*ini*_ (Fig 4A). Note that Fig 4 depicts the results at 0.75 m water depth (center of the second grid cell from the top) after 1000 years (symbols and solid lines) and after 100 years (dashed lines) of community development time. In many cases the results after 100 years are essentially the same as after 1000 years. Exceptions are the variance and the functional richness of unimodal trait distributions that are smaller after 1000 years than after 100 years simulation time.

In case of the development of bimodal trait distributions, simulation results obtained with RM and AM differ significantly with respect to several functionally relevant properties of the trait distributions despite the good agreement in the predicted total concentration of phytoplankton and the predicted variance of the trait distributions. In particular, the simulations with AM suggest that for *N*_*ini*_ < 45 mgP m^-3^ functional richness includes all trait groups in the entire trait space considered, whereas in RM only few trait groups survive 1000 yr simulation time (Fig 4D). Furthermore, AM assumes that the phytoplankton concentration distribution of the community has its maximum at the mean community trait, whereas RM predicts extinction of phytoplankton at intermediate trait values including the mean community trait, e.g. for *N*_*ini*_ < 50 mgPm^-3^ after 100 and more years of simulation time (Fig 2A–2D) and for *N*_*ini*_ = 30 mgPm^-3^ from 4 years simulation time onwards (Fig 2A). At 100 and 1000 years simulation time and at *N*_*ini*_ < 50 mgP m^-3^ the ratio of the concentration at mean trait to the maximum concentration *f*_*CTrav*/*Cmax*_ becomes essentially zero in case of RM (Fig 4G), whereas in all AM simulations *f*_*CTrav*/*Cmax*_ equals 1 because of the assumption that trait distributions are of Gaussian form.

Another characteristic of the trait distribution of the phytoplankton community important for trophic interactions is the trait at which the phytoplankton concentration distribution has its maximum. (*Tr*_*Cmax*_). In case of AM, *Tr*_*Cmax*_ is equal to the average community trait *Tr*_*av*_ (compare Fig 4E to 4B), whereas in case of RM this is only true for unimodal distributions but not for bimodal distributions developing in the simulations with *N*_*ini*_ < 50 mgP m^-3^ (Fig 4E). If RM predicts a bimodal trait distribution, *Tr*_*Cmax*_ is the trait of one of the co-existing trait groups. In contrast to functional richness the index of functional divergence *FD*_*80*_ of the trait distributions predicted by AM and RM agrees well not only in case of unimodal but also in case of bimodal trait distributions (Fig 4F). The only exception is at *N*_*ini*_ = 10 mgP m^-3^, for which RM predicts a very small divergence index (Fig 4F), because the phytoplankton concentration of one of the two co-existing trait groups is larger than 90% of the total phytoplankton concentration at this depth.

The general characteristics of the trait distributions, i.e. unimodal or bimodal, remain the same throughout the vertical water column and depend on *N*_*ini*_ (Fig 1). Total abundance decreases with water depth except very near the surface where *C*_*T*_ slightly increases with water depth (Fig 3A and 3E). The depth of the concentration maximum depends on *N*_*ini*_ and co-existing trait groups typically have their concentration maximum at different depths (see Figure B in S1 File). RM and AM provide similar vertical profiles of *C*_*T*_ in case of bimodal trait distributions (e.g. at *N*_*ini*_ = 35 mgP m^-3^, Fig 3A) and also in case of unimodal trait distributions (e.g. at *N*_*ini*_ = 50 mgP m^-3^, Fig 3E). In case of unimodal trait distributions average community trait and functional diversity index *FD*_*80*_ are essentially independent of water depth and have the same values for RM and AM (Fig 3F and 3G). In case of bimodal trait distributions average community trait value of master trait *H*_*I*_ decreases with increasing depth in both models (Fig 3B). In AM this change in average community trait with depth results from a shift of the Gaussian trait distribution in trait space. However, in case of RM only two trait groups co-exist that differ in their traits but the traits of the two groups do not change within the entire water column. In RM the shift in average community trait results from a change in the relative abundance of the two trait groups (Fig 3D). Hence, in the vertical dimension the trait at maximum abundance *Tr*_*Cmax*_ only shifts between two distinct trait values in RM, whereas in AM *Tr*_*Cmax*_ changes continuously with depth assuming intermediate values between the two trait values of the trait groups co-existing in RM (Fig 3B). Note that in AM *Tr*_*Cmax*_ = *Tr*_*av*_ and is therefore covered by the red line in Fig 3B.

**Fig 5:**
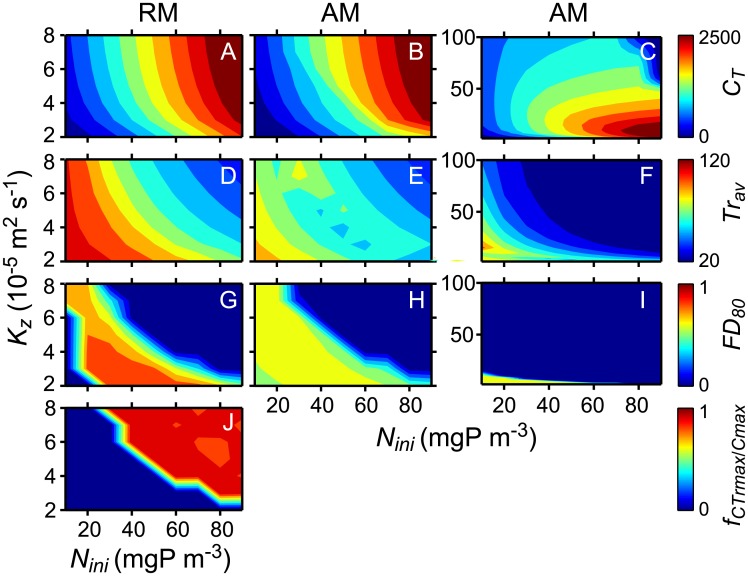
Properties of the trait distributions as function of nutrient enrichment and turbulent diffusivity. Depicted are the results at 0.75m depth after 100 yr development time. The units of *C*_*T*_ and *Tr*_*av*_ are mgC m^-3^ and μmol photons m^-2^ s^-1^, respectively.

Total phytoplankton concentration and the properties of the trait distribution of the phytoplankton community persisting after 100 years development time depend on *N*_*ini*_ and on *K*_*z*_ (Fig 5). *C*_*T*_ increases with increasing nutrient enrichment (increasing *N*_*ini*_) and with increasing mixing intensity (*K*_*z*_) for diffusivities below 10^−4^ m^2^s^-1^ (Fig 5A and 5B). At larger mixing intensities *C*_*T*_ declines with increasing *K*_*z*_ because intense mixing in the 50 m deep water column enhances light limitation (Fig 5C). This argument is supported by the decrease in the average community trait *H*_*I*_ with increasing mixing intensity (Fig 5F).

RM and AM predict similar changes of *C*_*T*_ with changes in *N*_*ini*_ and *K*_*z*_ (Fig 5A and 5B), whereas the predictions of *Tr*_*av*_ agree between the model approaches not for all combinations of *N*_*ini*_ and *K*_*z*_ but only when *N*_*ini*_ and *K*_*z*_ are high (Fig 5D, 5E, 5G and 5H). RM and AM predict sharp transitions from very low to high values of *FD*_*80*_ and these transitions occur at essentially the same combinations of *N*_*ini*_ and *K*_*z*_ in both models (Fig 5G and 5H). The exception are few simulations considering low *N*_*ini*_ and low *K*_*z*_ in which *FD*_*80*_ of RM is low and of AM is high. For all other combinations of *N*_*ini*_ and *K*_*z*_ at which the transitions of *FD*_*80*_ occur the concentration ratio *f*_*CTrav*/*Cmax*_ of the trait distributions predicted by RM changes from high to low values (Fig 5J) indicating a transition from a state with co-existence to a state with persistence of a single trait group. At large *K*_*z*_ *FD*_*80*_ is small (Fig 5G–5I) suggesting that low mixing intensities are required for evolutionary stable co-existence to occur. Similarly, nutrient enrichment leads to small *FD*_*80*_ suggesting that low rather than high nutrients support evolutionary stable co-existence, if phytoplankton differs only with respect to half saturation constants *H*_*I*_ and *H*_*N*_.

Similar results on selection and co-existence and on the agreement between RM and AM as above can also be obtained for another trade-off function between *H*_*I*_ and *H*_*N*_ and a smaller range of *H*_*I*_ (see Figures A and C in S1 File).

The conditions under which two competing phytoplankton species with fixed traits co-exist was additionally explored using a model considering only two-species (Figure D in S1 File) and compared to the conditions supporting evolutionary stable coexistence arising from selection in a multi-species community. At same trophic state, i.e. same *N*_*ini*_, the two species model predicts co-existence for several combinations of *H*_*I*_ (Figure D in S1 File). Furthermore, co-existence occurs for a wide range of trophic states, e.g. at *N*_*ini*_ from 10 to 70 mgPm^-3^ (Figure D in S1 File). In contrast, competition in the multi-species community model leads for each *N*_*ini*_ to the selection of a unique single trait group or a unique combination of two traits groups in case of evolutionary stable co-existence (Fig 4). At the same mixing conditions as in the two-species model (*K*_*z*_ = 5 10^−5^ m^2^ s^-1^) evolutionary stable co-existence occurs at a much narrower range of *N*_*ini*_, i.e. only if *N*_*ini*_ < 50 mgPm^-3^ (Figs 2 and 4) than in the two-species model.

## Discussion

This study investigates trait selection within a phytoplankton community limited by light and nutrients in partially mixed systems. Most model studies on competition of light and nutrient limited phytoplankton have been performed assuming fully mixed systems (e.g.[5,6]) or considered the competition between two prescribed species (e.g. [7,25]). In the following we first discuss our results from the community resolving trait-based model RM with respect to trait selection by competition within a phytoplankton community and the development of evolutionary stable co-existence. Afterwards, the potential and limitations of aggregated trait based models with adaptive traits to contribute to an understanding of community dynamics are discussed.

### Trait selection and long-term co-existence

The phytoplankton community considered in RM consists of a large number of species such that the available trait space of the master trait is essentially covered continuously. Trait selection and co-existence result from competition within this community for the two limiting resources, nutrients and light. Competition lead either to evolutionary stable co-existence of essentially two trait groups and the development of bimodal trait distributions, or to the extinction of all trait groups but one and unimodal trait distributions (Fig 1). The transition from the development of bimodal to unimodal trait distributions with nutrient enrichment, i.e. an increase in *N*_*ini*_, and with an increase in *K*_*z*_ is associated with a strong change in *FD*_*80*_ from values close to one to values close to zero (Fig 4F) and an opposite change in *f*_*CTrmax*/*Cmax*_ (Fig 4G). Changes of *FD*_*80*_ and *f*_*CTrmax*/*Cmax*_ in opposite directions thus suggest transitions of the final state, i.e. between the co-existence of two and the persistence of one trait group. In few RM simulations with low *N*_*ini*_ and low *K*_*z*_ one of the two co-existing trait groups had concentrations below 10% of *C*_*T*_ leading to very small *FD*_*80*_ (Figs 4F and 5G). In these cases, low *FD*_*80*_ are associated with low concentration ratios *f*_*CTrmax*/*Cmax*_ (Figs 4G and 5J) the latter correctly indicating co-existence despite the low *FD*_*80*_. Note that in these cases *FD*_*80*_ of the distributions simulated with AM are large (Figs 4F and 5H) and thus are a better indicator of co-existence than *FD*_*80*_ of the simulated distributions obtained with RM.

In fully mixed systems co-existence of light and nutrient limited phytoplankton requires differences in resource consumption [5,6]. Our simulation results show that in partially mixed systems differences in resource consumption are not required but that differences in specific production, i.e. due to differences in half saturation constants *H*_*I*_ and *H*_*N*_, are sufficient to support evolutionary stable co-existence of phytoplankton (Fig 1A–1C). In our simulations with RM the selection process within a community consisting of numerous groups with different trait combinations reduces the functional richness of the original community and leads to the persistence of one or the coexistence of two very narrow trait groups. This result is consistent with results from an eco-evolutionary model with adaptive dynamics simulating monomorphic species of phytoplankton using essentially the same phytoplankton model as in the study here and predicting evolutionary stable co-existence of singular trait groups [8].

In case of the development of evolutionary stable coexistence, competition within the community leads to selection of one trait groups that has comparatively low *H*_*I*_ supporting efficient use of light at low light intensities in deeper waters where the nutrient concentrations are typically higher than in the surface waters. The other co-existing trait group selected sacrifices good performance at low light conditions for good performance at low nutrient levels (*H*_*I*_ is large and *H*_*N*_ low). The latter trait group benefits from high light intensities at the lake surface but must be efficient at low nutrient levels because nutrient concentrations decrease towards the surface due to nutrient uptake by the phytoplankton in deeper waters. Note that the co-existing trait groups are not separated in the vertical dimension but also co-exist at the same water depths (Fig 3D and Figure B in S1 File). The conditions at a specific depth of co-existences may not support persistence of both trait groups but vertical transport of the trait groups from other depths with higher net production may compensate for local losses. Thus transport from different niches in the vertical water column enables co-existence although the competing species do not differ in their resource consumption. According to [31] and [32] co-existence of a superior and an inferior competitor is possible if spatial niches exist and the inferior competitor is the more fugitive species, i.e. has a higher colonization rate. In our model all individuals have the same spatial mobility, i.e. the same sinking rate. However, the vertical distributions of the populations with different traits differ and therefore the vertical transport due to turbulent diffusion differs between trait groups. Hence, differences in the spatial distribution of the populations causing differences in diffusive fluxes rather than differences in individual mobility support co-existence of our phytoplankton community differing only with respect to half saturation constants for light and nutrients. Co-existence of more phytoplankton species than limiting resources (see [8]) may also be explained by the combination of spatial niches with differences in the transport of species due to differences in their vertical distribution patterns. Our results thus suggest that conclusions on the conditions for co-existence of phytoplankton derived from analyses of fully mixed systems as in [5] or [6] may not necessarily be directly applicable to partially mixed systems that allow for variation in the habitat conditions in the vertical dimension.

According to the results on trait selection from RM, functional divergence *FD*_*80*_ (Fig 4G), i.e. the trait difference of the co-existing species, increases with decreasing *N*_*ini*_ (Figs 4G and 2A–2D). This result suggests that reducing nutrients may support communities consisting of species with extreme traits.

The trophic state *N*_*ini*_ under which two competing species can co-exist depends not only on the trade-off function between trades but also on the specific values of the master trait available for the two species (Figure D in S1 File). This suggests that the eco-physiological limits of the trait space for a community consisting of multiple species with different traits but the same trade-offs, will influence the conditions under which co-existence can occur.

### Limitations and potential of aggregated trait based approaches with adaptive traits

The above conclusions were derived from simulation results obtained with RM, i.e. a model that resolves individual trait groups. In the following we address the question, whether and in which way aggregated trait based approaches can support the investigation of the response of community dynamics to changing conditions.

AM and RM agree very well for unimodal trait distributions, not only after long simulation times (e.g. Fig 4 for *N*_*ini*_ ≥ 50 mg m^-3^) but also during the dynamic development of phytoplankton communities from the initial trait distribution to the final state (Fig 2) and in the vertical dimension (Fig 3E–3H). This excellent agreement between AM and RM indicates that the mathematical approach and numerical implementation of AM designed to simulate aggregated properties of phytoplankton trait distributions in vertically resolved systems is adequate.

At low values of *N*_*ini*_ the trait distributions change their shape from normal to non-normal distributions and eventually become bimodal (e.g. Fig 1A–1C and RM in Fig 2A–2D). Although in these cases the assumption of normal trait distributions employed in the solution of AM is not valid, the estimates of total concentration and the functional divergence of the trait community agree well between AM and RM (Fig 4A and 4F). In contrast, the average community trait is shifted to lower values in AM compared to RM (e.g. Figs 4B and 3B), and functional richness and the concentration ratio *f*_*CTrav*/*Cmax*_ of the trait distributions simulated with AM and RM, respectively, strongly differ after long simulation times (Fig 4D and 4G). The disagreements reflect the differences between the bimodal trait distributions obtained with RM after long simulation times and the trait distributions inferred from the statistical properties predicted by AM using the underlying assumption of normal trait distributions (Fig 2A–2D).

Thus, although some of the statistical properties of the trait distributions predicted from RM and AM are similar, the trait distributions themselves differ in functionally important aspects which may have severe implications for the understanding of e.g. trophic interactions between zooplankton and the phytoplankton community. Assuming normal trait distributions as in AM, an average community trait *Tr*_*av*_ at intermediate trait values suggests that phytoplankton concentrations at intermediate trait values are large and the phytoplankton community thus supports zooplankton feeding on phytoplankton with intermediate traits. Note, that traits typically correlate with size of the organisms [33] and availability of phytoplankton at intermediate traits thus suggests availability of phytoplankton at intermediate size. Further, the abundances of specific zooplankton groups as well as overall zooplankton trait distributions are closely coupled to the traits of phytoplankton [34]. However, the results from RM indicate, that *Tr*_*av*_ at intermediate trait values may also result from the abundance of two distinct trait groups at the outer range of the trait space and that phytoplankton with trait values *Tr*_*av*_ have become extinct and are not available for grazing. This suggests that in case of bimodal trait distributions obtained from RM simulations, the corresponding AM simulations would erroneously predict high abundances at intermediate trait values, which should support different zooplankton taxa and/or zooplankton trait distributions compared to RM simulations. Likewise AM simulations erroneously suggest different niches for zooplankton with depth as they assume normal trait distributions and predict continuous shifts in the average phytoplankton community trait with increasing depth (Fig 3B). RM simulations clarify that this shift in average community trait results from changes in the relative abundance of two persisting trait groups.

Aggregated trait based modelling approaches may provide reasonable approximations of statistical properties of the trait distribution of the community, but the statistical properties calculated with AM, i.e. *C*_*T*_, *Tr*_*av*_ and *Tr*_*var*_, do not provide sufficient information to infer unique trait distributions and the availability of phytoplankton at certain trait values. Further, *Tr*_*var*_ may be an important characteristic of the trait distribution relevant for the prediction of the change in average community trait, but it does not correlate with functional richness. Hence, even if AM and RM may provide similar results on *C*_*T*_, *Tr*_*av*_ and *Tr*_*var*_, these statistical properties do not allow reliable conclusions on community dynamics, community diversity, competitive abilities and selection, or trophic interactions between communities. The comparison of the predictions from RM and AM and the properties of the trait distributions simulated with RM point to a rather general difficulty of aggregated trait based approaches: The assessment of a few statistical properties of trait distributions. i.e. *C*_*T*_, *Tr*_*av*_ and *Tr*_*var*_, apparently is not a sufficient basis for an understanding of community dynamics if the communities do not have normal trait distributions but develop multimodal trait distributions.

**Fig 6:**
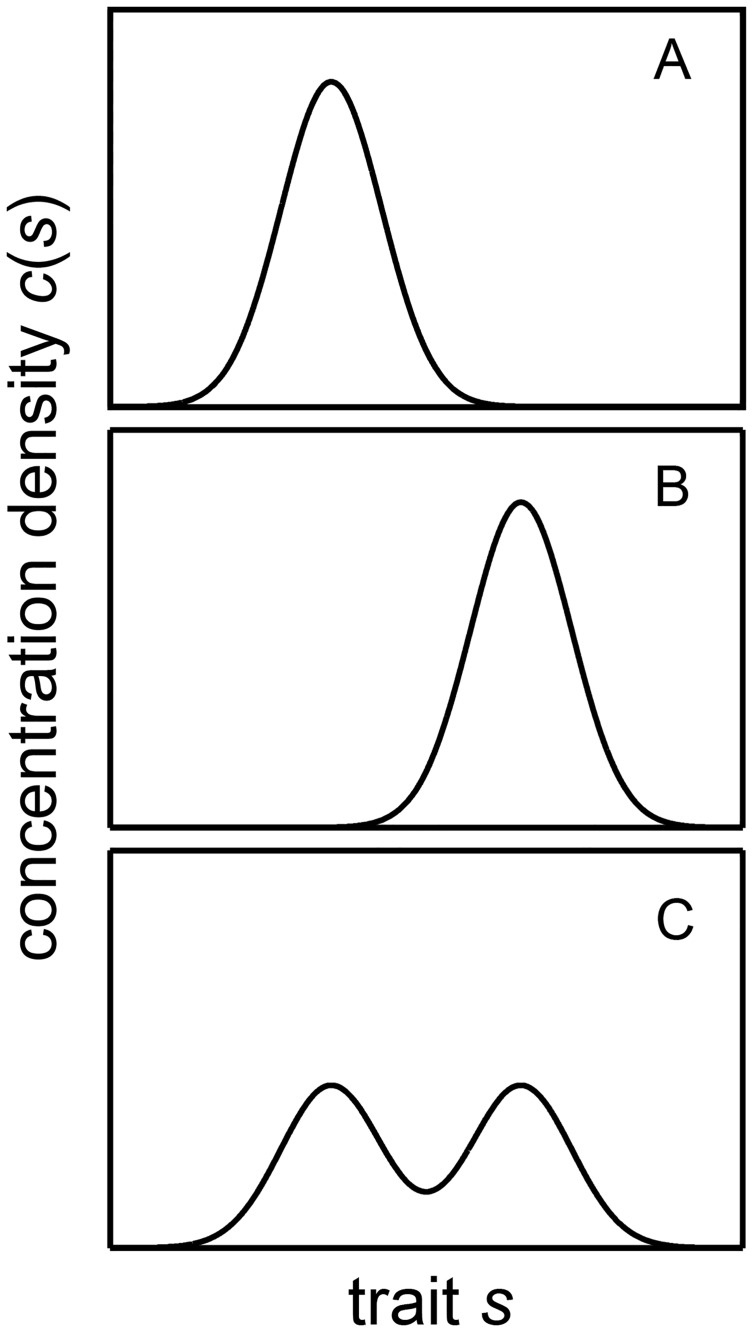
Illustration of the generation of bi-modal trait distributions by mixing: Two communities that are characterized by the same master trait have trait distributions of Gaussian form with the same concentration *C*_*T*_ and variance *Tr*_*var*_, but differ in the mean community trait *Tr*_*av*_ (A, B). Mixing of these two communities results in a community that has a bi-modal distribution of the master trait (C).

There is no observational evidence demonstrating normally distributed or at least unimodal trait distributions in phytoplankton. Furthermore, in partially mixed environments multimodality of trait distributions of plankton communities can easily arise and may be a common feature because in systems with resource gradients in which trait distributions vary spatially, mixing does not conserve the shape of trait distributions or their unimodality. This effect of mixing is illustrated in Fig 6: Consider two water masses containing phytoplankton communities that have normal trait distributions with respect to one trait. For simplicity we assume that both communities have the same total concentration and that their trait distributions have the same variance but differ in their average community trait (Fig 6A and 6B). Mixing of the two water masses results in a community that is characterized by a bimodal trait distribution (Fig 6C). This illustration suggests that the assumption that mixing restores unimodal trait distributions [35] may not necessarily be justified because mixing has the potential to generate multimodal from unimodal distributions. The requirement of unimodal trait distributions in AM may thus be particularly problematic in systems in which trait distributions differ in space and partial mixing is important.

The premise in trait based ecology is that environmental conditions are reflected in the trait distributions of a community. Hence, in environments with spatial resource gradients trait distributions should differ in space. In case of phytoplankton, trait distributions with respect to light and nutrient utilization, e.g. the half saturation constant of light and nutrients, may differ not only in the vertical but also in the horizontal dimension because shore regions are typically enriched in nutrients, particularly close to inflows, and/or are more turbid than the open waters. Because phytoplankton community traits can be expected to vary in space and to be mixed by horizontal turbulent diffusion as in Fig 6, unimodal trait distributions in phytoplankton communities may be an exception rather than common in natural systems.

The discussion above suggests that non-normal and even bimodal trait distributions of light and nutrient limited phytoplankton communities may be common in partially mixed systems. Likewise disruptive selection caused by predators may also result in bimodal trait distributions of phytoplankton depending on the specific trade-off between phytoplankton growth rates and edibilities [18]. In all these cases, phytoplankton trait distributions inferred from the results of AM can be misleading in studies of trophic interactions, especially if zooplankton grazing rates depend on phytoplankton and zooplankton traits and the interaction is described based on average community traits. However, this conclusion does not imply that AM cannot support the analysis of community dynamics. In our partially mixed water column competition and selection in a light und nutrient limited phytoplankton community results in the development of evolutionary stable co-existences of essentially two trait groups or in the persistence of one trait group depending on environmental conditions and phytoplankton traits. Assessment of the conditions for co-existence can be based on model predictions of the functional divergence *FD*_*80*_ which are similar in AM and RM simulations. *FD*_*80*_ determined for simulation results from AM can therefore be used to indicate whether the final state implies persistence of a single trait group or co-existence of two trait groups. Because the computational time required for simulations with aggregate models are much shorter than for models resolving numerous species [35], the application of AM can be an efficient way to assess the combination of environmental conditions and phytoplankton traits under which co-existence may occur (e.g. Fig 5H and 5I and Figure C panel F in S1 File). Furthermore, if unimodal trait distributions develop the effect of environmental conditions on the selected traits can be assessed using AM, as e.g. in Fig 5 illustrating that at large mixing intensities the average community trait *H*_*I*_ decreases with increasing *K*_*z*_.

A common difficulty in AM models is that trait variance and thus diversity vanishes with simulation time [36] if the species model underlying the AM leads to selection of a single species. This also implies that the diversity in the corresponding RM vanishes. As a solution to the difficulty of decreasing diversity in plankton models [36] have suggested to increase diversity, i.e. trait variance, by introducing diffusion in trait space. However, diffusion in trait space does not have a proper expression in the processes considered in the individual models forming the basis for the AM. Furthermore, the increase in variance due to trait diffusion in unimodal trait distributions cannot be distinguished from an increase in variance due to the development of bimodal trait distributions. Hence, the development of trait variance in AM models is not a reliable measure of trait diversity.

Concluding the discussion, our results confirm that in partially mixed systems evolutionary stable co-existence of light and nutrient limited phytoplankton can occur if phytoplankton differs only with respect to specific production. Differences in resource consumption are not required for co-existence to occur. However, if mixing intensities are large differences in specific production appear insufficient to support co-existence. The two model approaches RM and AM agree well if trait distributions are unimodal but differ if trait distributions become bi-modal, as was the case in our simulations leading to evolutionary stable co-existence of two trait groups. In systems with spatial resource gradients that are partially mixed multimodality may easily develop. In these cases AM may be misleading with respect to community dynamics but can support exploration of conditions and parametrizations at which the system undergoes transitions, e.g. from the persistence of a single to co-existence of more than one trait group.

## Supporting information

S1 File Supporting information containing 6 parts. Appendix A in S1 File provides adetailed description of the aggregated trait-based model, Table A in S1 File lists model variables and parameters. Figure A in S1 File depicts the trade-off between the half saturation constants *H*_*N*_ and *H*_*I*_. Figure B in S1 File shows different properties of the RM-simulated trait distributions in the vertical dimension. Figure C in S1 File presents model results considering a smaller range of *H*_*I*_ and *H*_*N*_ and a different trade-off function than in the main text and Figure D in S1 File shows results from a two species model.
(PDF)
