# Supplementary material for: Trait selection and co-existence of phytoplankton in partially mixed systems: Trait based modelling and potential of an aggregated approach
Source: PLoS One. 2018 Mar 22;13(3):e0194076. doi: 10.1371/journal.pone.0194076 (PMC5863978; doi:10.1371/journal.pone.0194076)
Supplement: S1 File — Appendix A in S1 File provides adetailed description of the aggregated trait-based model, Table A in S1 File lists model variables and parameters. Figure A in S1 File depicts the trade-off between the half saturation constants HN and HI. Figure B in S1 File shows different properties of the RM-simulated trait distributions in the vertical dimension. Figure C in S1 File presents model results considering a smaller range of HI and HN and a different trade-off function than in the main text and Figure D in S1 File shows results from a two species model. (PDF) [file pone.0194076.s001.pdf]

Supporting Information for

"Trait selection and co-existence of phytoplankton in partially mixed systems: Trait based modelling and potential of an aggregated approach"

Frank Peeters and Dietmar Straile

Limnological Institute, University of Konstanz, Mainaustrasse 252, D-78464 Konstanz, Germany

The Supporting Information includes six parts:

Appendix A: Detailed description of the aggregated trait-based model

Table A: Tables A.1 and A.2 provide model variables and parameters, respectively

Figure A: Depiction of the trade-off between the half saturation constants H_N and H_I

Figure B: Properties of the RM-simulated trait distributions in the vertical dimension

Figure C: Model results considering a smaller range of H_I and H_N. and a different trade-off function than in the main text

Figure D: Results from a two species model

Appendix A: Detailed description of the aggregated trait-based model

The model employed in this study assumes that without transport the concentration of an individual species C_i changes with time according to:

$$\frac{dC_i}{dt} = r\left(s_i, I, N\right) \cdot C_i \qquad \text{(S1)}$$

r is the net growth rate as function of the two limiting resources light, I, and nutrients, N. s_i is the value of the master trait s of species i. Assuming a continuous trait space the community consists of an indefinite number of individual species. The community can then be described by a trait based approach using a concentration density c with respect to trait s. The total concentration C_T and the first and second moment of the trait distribution M_1 and M_{11} of the community can be determined by integration.

$$\begin{aligned} C_T &= \int c(s) \cdot ds \\ M_1 &= \int s \cdot c(s) \cdot ds \\ M_{11} &= \int s^2 \cdot c(s) \cdot ds \end{aligned} \qquad \text{(S2)}$$

The central moments of the distributions can be calculated from:

$$\begin{aligned} s_{av} &= \frac{M_1}{C_T} \\ s_{\mathrm{var}} &= \frac{M_{11}}{C_T} - {s_{av}}^2 \end{aligned}, \qquad \text{(S3)}$$

and s_{av} is the center of mass of the trait distribution which is called the average community trait and s_{var} is the variance of the trait distribution around the average community trait and called the trait variance.

Assuming continuous trait space the set of differential equations describing the individual species with different trait values are combined to provide a differential equation for the total concentration of the community.

$$\frac{dC_T}{dt} = \int \frac{dc(s)}{dt} \cdot ds = \int r\left(s, I, N\right) \cdot c(s) \cdot ds \tag{S4}$$

The growth rate of each species is approximated by a Taylor expansion around the growth rate at the average community trait considering terms up to the second order:

$$\begin{aligned} r(s) &= r(s_{av}) + \left(s - s_{av}\right) \cdot \left.\frac{\partial r}{\partial s}\right|_{s=sav} + \frac{1}{2}\left(s - s_{av}\right)^2 \cdot \left.\frac{\partial^2 r}{\partial s^2}\right|_{s=sav} \\ r_m &= r(s_{av}) = r\left(M_1/C_T, I, N\right) \end{aligned} \tag{S5}$$

The integral in eq. S4 leads to:

$$\frac{dC_T}{dt} \approx r_m \cdot C_T \quad + \frac{1}{2} \cdot \left(M_{11} - \frac{M_1^2}{C_T}\right) \cdot \left.\frac{\partial^2 r}{\partial s^2}\right|_{M_1/C_T} \tag{S6}$$

Note that the Taylor expansion requires derivatives of the growth rate with respect to the traits investigated. In our study we consider a trade-off between the half saturation constants for light, H_I, and nutrients, H_N and use H_I as master trait. Because Liebig's minimum law cannot be

differentiated with respect to the half saturation constants we employ in our study a product law for the description of co-limitation of phytoplankton by light and nutrients.

Production leads to the uptake of dissolved nutrients and the loss of dissolved nutrients depends on the specific rate of nutrient uptake q by each species. Considering the concentration density *c(s)* instead of individual species and using a Taylor approximation as above, the rate of change in dissolved nutrients can be described as:

$$
\begin{aligned}
\frac{dN}{dt} &= -\int q(s,I,N)\cdot c(s)\cdot ds \\
\frac{dN}{dt} &\approx -\left[q_m \cdot C_T \quad + \quad \frac{1}{2}\cdot\left(M_{11} - \frac{M_1^{\,2}}{C_T} \right) \right] \cdot \left. \frac{\partial^2 q}{\partial s^2} \right|_{M_1/C_T} \\
q_m &= q\left(M_1/C_T\,, I, N\right)
\end{aligned}
\qquad \text{(S7)}
$$

The uptake of nutrients is proportional to the net production of each species and depends also on the stoichiometry of the species. In our parametrization we assume that all phytoplankton species have the same stoichiometry. Then the specific rate of nutrient uptake rate is proportional to the specific net production, i.e. $q(s,I,N) = \gamma \cdot r(s,I,N)$ and γ is nutrient uptake per unit biomass produced by the algae. If the stoichiometry is the same for all species the total rate of nutrient consumption is proportional to the total biomass production.

$$
\frac{dN}{dt} = -\gamma \cdot \frac{dC_T}{dt} \qquad \text{(S8)}
$$

Note, that the differential equations for C_T and N include first and second moments of the trait distributions. As the community composition changes with time also the moments of the trait

distribution are time dependent. Thus the solution of eqs. S6 and S8 requires also the simulation of the first and the second moment of the trait distribution. Differential equations for M_1 and M_{11} can be derived in a similar manner as for C_T. However, these equations involve third and fourth moments of the trait distribution leading to a closure problem.

This closure problem has been solved by making assumptions on the distribution of the traits around the average community traits. Wirtz and Eckhardt (1996), Merico et al. (2009; 2014), and Terseleer et al. (2014) assume that the trait distributions are of Gaussian form, whereas Bruggeman (2009) assumes log-normal distributions. Both approaches imply that the trait distributions are unimodal. Norberg et al. (2001) and Savage et al. (2007) use as closure scheme that requires a fully resolved model from which relationships between 3rd and 4th order moments and lower order moments of the trait distribution are estimated numerically. In this case, it is not possible to derive a unique trait distribution from the results of the aggregated trait based model.

Here we adopted the approach of Wirtz and Eckhardt (1996) and Merico et al. (2009) assuming that the trait distributions are of Gaussian form. The resulting differential equations for the first and second moment of the trait distributions are:

$$\begin{aligned}
\frac{dM_1}{dt} &\approx r_m \cdot M_1 + \left(M_{11} - \frac{M_1^2}{C_T}\right) \cdot \left.\frac{\partial r}{\partial s}\right|_{M_1/C_T} + \frac{1}{2}\cdot\left(\frac{M_1 \cdot M_{11}}{C_T} - \frac{M_1^2}{C_T^2}\right) \cdot \left.\frac{\partial^2 r}{\partial s^2}\right|_{M_1/C_T} \\
\frac{dM_{11}}{dt} &\approx r_m \cdot M_{11} + 2\cdot\left(\frac{M_1 \cdot M_{11}}{C_T} - \frac{M_1^3}{C_T^2}\right)\cdot \left.\frac{\partial r}{\partial s}\right|_{M_1/C_T} + \frac{1}{2}\cdot\left(2\frac{M_1^4}{C_T^3} - 5\frac{M_1^2 \cdot M_{11}}{C_T^2} + 3\frac{M_{11}^2}{C_T}\right)\cdot\left.\frac{\partial^2 r}{\partial s^2}\right|_{M_1/C_T}
\end{aligned} \quad \text{(S9)}$$

In a vertical water column, phytoplankton concentrations, light intensity and nutrients depend on water depth and vertical transport due to turbulent diffusion affects the vertical distribution of phytoplankton and nutrients. In addition sedimentation leads to advective transport

of phytoplankton. Including the vertical dimension in the model equations above, assuming that phytoplankton differs only with respect to H_I and H_N and using H_I as master trait s, the full model used in this study becomes:

$$
\begin{aligned}
\frac{\partial C_T}{\partial t} &\approx r_m \cdot C_T + \frac{1}{2}\cdot\left(M_{11} - \frac{M_1^{\,2}}{C_T}\right)\cdot\left.\frac{\partial^2 r}{\partial H_I^{\,2}}\right|_{M_1/C_T} - v\frac{\partial C_T}{\partial z} + \frac{\partial}{\partial z}\left(K_z\frac{\partial C_T}{\partial z}\right)\\
\frac{\partial M_1}{\partial t} &\approx r_m \cdot M_1 + \left(M_{11} - \frac{M_1^{\,2}}{C_T}\right)\cdot\left.\frac{\partial r}{\partial H_I}\right|_{M_1/C_T} + \frac{1}{2}\cdot\left(\frac{M_1\cdot M_{11}}{C_T} - \frac{M_1^{\,2}}{C_T^{\,2}}\right)\cdot\left.\frac{\partial^2 r}{\partial H_I^{\,2}}\right|_{M_1/C_T} - v\frac{\partial M_1}{\partial z} + \frac{\partial}{\partial z}\left(K_z\frac{\partial M_1}{\partial z}\right)\\
\frac{\partial M_{11}}{\partial t} &\approx r_m \cdot M_{11} + 2\cdot\left(\frac{M_1\cdot M_{11}}{C_T} - \frac{M_1^{\,3}}{C_T^{\,2}}\right)\cdot\left.\frac{\partial r}{\partial H_I}\right|_{M_1/C_T} + \frac{1}{2}\cdot\left(2\frac{M_1^{\,4}}{C_T^{\,3}} - 5\frac{M_1^{\,2}\cdot M_{11}}{C_T^{\,2}} + 3\frac{M_{11}^{\,2}}{C_T}\right)\cdot\left.\frac{\partial^2 r}{\partial H_I^{\,2}}\right|_{M_1/C_T} - v\frac{\partial M_{11}}{\partial z} + \frac{\partial}{\partial z}\left(K_z\frac{\partial M_{11}}{\partial z}\right)\\
\frac{\partial N}{\partial t} &\approx -\gamma\cdot\left(r_m \cdot C_T + \frac{1}{2}\cdot\left(M_{11} - \frac{M_1^{\,2}}{C_T}\right)\cdot\left.\frac{\partial^2 r}{\partial H_I^{\,2}}\right|_{M_1/C_T}\right) + \frac{\partial}{\partial z}\left(K_z\frac{\partial N}{\partial z}\right)\\
\frac{dN_s}{dt} &= \sum v\cdot\gamma\cdot C_T\left(z_{\max}\right) - m\cdot N_s\\
I(z) &= I_0\exp\left(-\int_0^z \sum\left(k\cdot C_T(z) + k_{bg}\right)dz\right)\\
r\left(H_I, I(z), N(z)\right) &= \mu_{\max}\frac{I(z)}{H_I + I(z)}\cdot\frac{N(z)}{f(H_I) + N(z)} - l_{bg}\\
r_m &= r\left(M_1(z)/C_T(z), I(z), N(z)\right)
\end{aligned}
$$

(S10)

Light intensity deceases exponentially with increasing depth whereby light absorption depends on the phytoplankton concentration in the water column. As bottom boundary condition for dissolved nutrients we used the flux of nutrients released at rate m from a nutrient pool N_s stored in the sediments. The concept of the sediment compartment was adopted from Jäger et al. (2010). All phytoplankton species are assumed to have the same specific maximum growth rate μ_{max}, specific loss rate l_{bg}, specific light extinction coefficient k, nutrient to carbon ratio γ, and sedimentation velocity v. Phytoplankton differs only in the half saturation constants for light H_I and nutrients H_N. The trade-off function between H_I and H_N is modelled as power function described by $\ln(H_N) = a + b\ln(H_I)$.

References:

Bruggeman, J. 2009. Succession in plankton communities - A trait-based perspective. Dep. Theor. Biol. **PhD thesis**: 158.

Jäger, C. G., S. Diehl, and M. Emans. 2010. Physical determinants of phytoplankton production, algal stoichiometry, and vertical nutrient fluxes. Am. Nat. **175**: E91–E104.

Merico, A., G. Brandt, L. S. Smith, and M. Oliver. 2014. Sustaining diversity in trait-based models of phytoplankton communities. Front. Ecol. Evol. **2**: 1–8.

Merico, A., J. Bruggeman, and K. Wirtz. 2009. A trait-based approach for downscaling complexity in plankton ecosystem models. Ecol. Modell. **220**: 3001–3010.

Norberg, J., D. P. Swaney, J. Dushoff, J. Lin, R. Casagrandi, and S. A. Levin. 2001. Phenotypic diversity and ecosystem functioning in changing environments: A theoretical framework. Proc. Natl. Acad. Sci. **98**: 11376–11381.

Ryabov, A. B., and B. Blasius. 2014. Depth of the Biomass Maximum Affects the Rules of Resource Competition in a Water Column. Am. Nat. **184**: E132–E146.

Savage, V. M., C. T. Webb, and J. Norberg. 2007. A general multi-trait-based framework for studying the effects of biodiversity on ecosystem functioning. J. Theor. Biol. **247**: 213–229.

Terseleer, N., J. Bruggeman, C. Lancelot, and N. Gypens. 2014. Trait-based representation of diatom functional diversity in a plankton functional type model of the eutrophied southern North Sea. Limnol. Oceanogr. **59**: 1958–1972.

Wirtz, K. W., and B. Eckhardt. 1996. Effective variables in ecosystem models with an application to phytoplankton succession. Ecol. Modell. **92**: 33–53.

Yoshiyama, K., J. P. Mellard, E. Litchman, and C. A. Klausmeier. 2009. Phytoplankton Competition for Nutrients and Light in a Stratified Water Column. Am. Nat. **174**: 190–203.

Table A: Model variables (Table A.1) and parameters (Table A.2)

Table A.1: Definitions of variables in RM and AM.

Variable	Value	Definition and unit
C_i		concentration of species *i* (mg C m^{-3})
C_T		Total phytoplankton concentration $C_T = \sum_i C_i$ (mg C m^{-3})
N	10 to 90[b]	Concentration of dissolved nutrients (mg P m^{-3})
N_s	0[a]	Pool of nutrients in the sediments (mg P m^{-2})
I		Light intensity (µmol photons m^{-2} s^{-1})
$p_{spec,i}$		Specific production rate of species i (d^{-1})
r_i		Net growth rate of species i (d^{-1})
t		Time (d)
z		Water depth below the lake surface (m).
$c(s)$		Concentration density of phytoplankton with respect to trait *s*. Here, *s* is the half saturation of light and the units of $c(H_I)$ are (mg C m^{-3}) (µmol photons m^{-2} s^{-1})$^{-1}$
C_T		Total phytoplankton concentration $C_T = \int c(s)ds$ (mg C m^{-3})
M_1		First moment $M_1 = \int s \cdot c(s)ds$ of the phytoplankton community with respect to trait *s*. Here $s = H_I$ and the units of M_1 are (mg C m^{-3}) (µmol photons m^{-2} s^{-1})
M_{11}		Second moment $M_{11} = \int s^2 \cdot c(s)ds$ of the phytoplankton community with respect to trait *s*. Here $s = H_I$ and the units of M_{11} are (mg C m^{-3}) (µmol photons m^{-2} s^{-1})^2

[a] Initial values

[b] Range of initial conditions examined.

Table A.2: Definitions of parameters and parameter values.

Parameter	Value	Definition and unit
K_z	$2 \cdot 10^{-5}$ to $\cdot 10^{-3}$	Turbulent-diffusion coefficient ($m^2\ s^{-1}$)
I_0	300	Light intensity at the surface (μmol photons $m^{-2}s^{-1}$)
k_{bg}	0.1	Background light-attenuation coefficient (m^{-1})
k	0.0003	Specific light-attenuation coefficient of algal biomass (m^2 mgC^{-1})
H_I	20 to 120	Half-saturation constant of light-dependent algal production (μmol photons $m^{-2}s^{-1}$)
H_N	0.01 to 5	Half-saturation constant of algal nutrient uptake ($mgPm^{-3}$)
a , b	12, -3.4684	Coefficients of the power function describing the trade-off between H_I and H_N : $\ln(H_N) = a + b \cdot \ln(H_I)$ which is equivalent to $H_N = c \cdot H_I^b$ with $c = e^a$. H_I and H_N have the units as above.
μ_{max}	1	Maximum specific algal production rate (d^{-1})
γ	0.025	Stoichiometry: phosphorus to carbon ratio of phytoplankton (mgP/mgC)
l_{bg}	0.25	Specific algal loss rate (d^{-1})
m	0.02	Specific mineralization rate of nutrients in the sediments (d^{-1})
v	0.25	Algal sinking velocity ($m\ d^{-1}$)
$z_{\max}$	50	z at maximum water column depth (m)

Figure A: Depiction of the trade-off between the half saturation constants H_N and H_I.

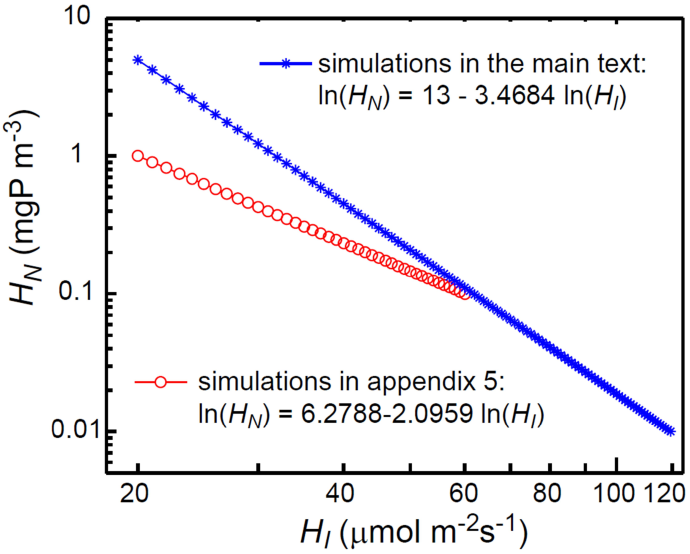

Figure A: The trade-off between the half saturation constants H_N and H_I. Depicted is the trade-off function used in the simulations described in the main text (blue) and the trade-off function used in the simulations presented in the S5 appendix (red). The symbols indicate the traits of the individual trait groups considered in RM. In all simulations with RM trait groups differ in H_I by 1 µmol m^{-2} s^{-1}. In the simulations with RM in the main text 101 trait groups were considered and H_I ranged between 20 and 120 µmol m^{-2} s^{-1}. The simulations in S5 appendix are based on the 41 trait groups with H_I ranging between 20 and 60 µmol m^{-2} s^{-1} depicted as red symbols here.

Figure B: Properties of the RM-simulated trait distributions in the vertical dimension

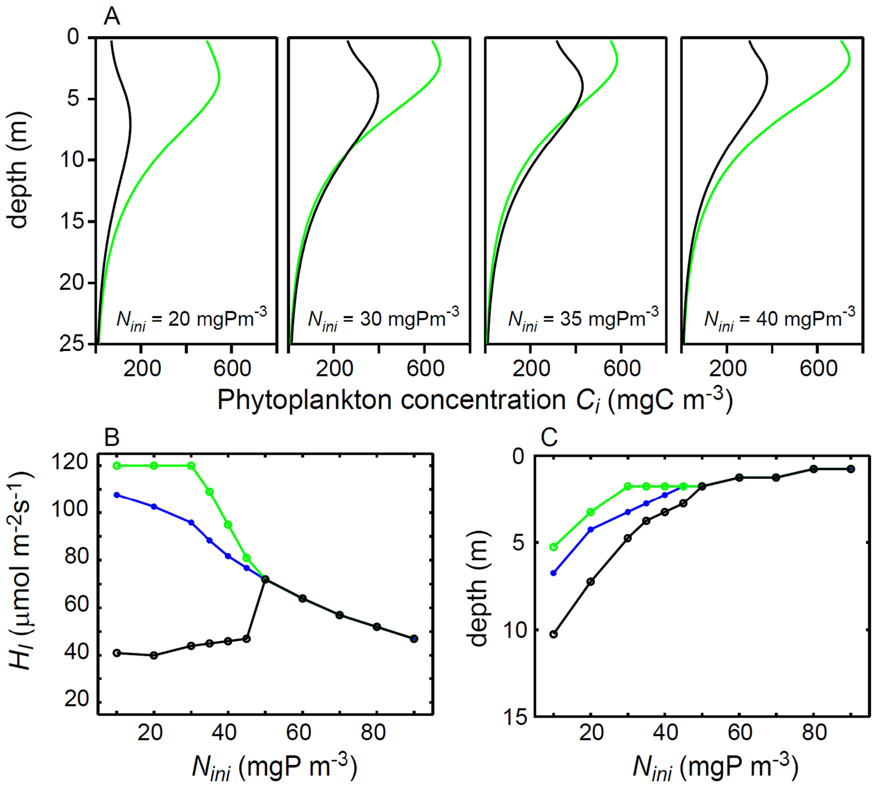

Figure B: Properties in the vertical dimension of the trait distributions simulated with RM using the same parameters as in the main text. (A) Vertical profiles of the two trait groups selected after 1000 yr simulation time under different nutrient enrichments. (B) Tr_{av} (blue) and the traits of the selected trait groups (black and green) at maximum concentration in these vertical distributions. (C) Depth of the maximum in the vertical distribution of C_T (blue) and of the selected trait groups (black and green) as function of nutrient enrichment. In all simulations turbulent diffusion was $K_z = 5 \cdot 10^{-5}$ m^2 s^{-1} and the simulated time period was 1000 yrs.

Figure C: Model results considering a smaller range of H_I and H_N. and a different trade-off function than in the main text

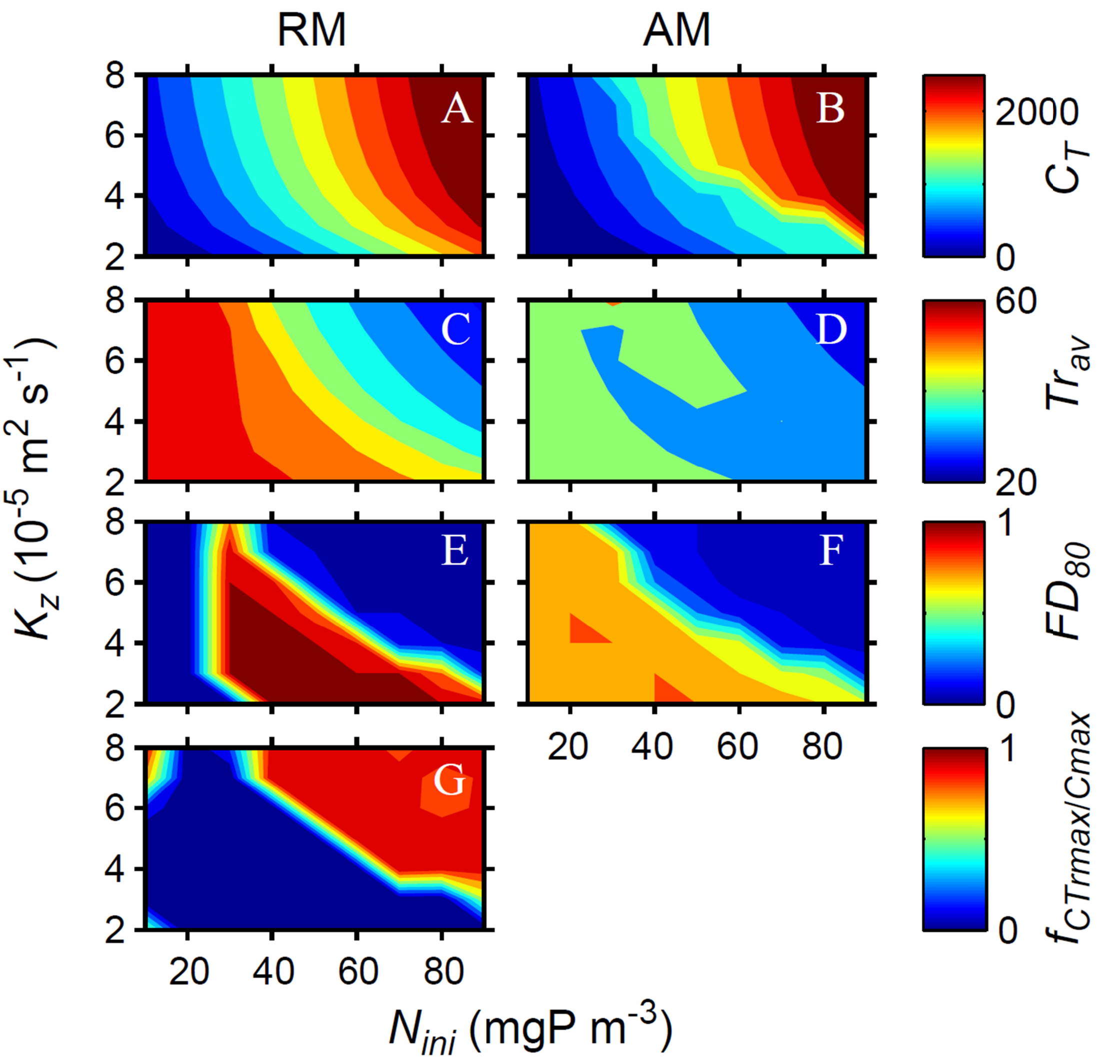

Figure C: Properties of the trait distributions as function of nutrient enrichment and turbulent diffusivity as in Figure 5 but for a different trade-off function and a smaller range of H_I and H_N. The trade-off between the half saturation constants is $\ln(H_N) = 6.2788\text{-}2.0959 \ln(H_I)$ (Fig. S1). In RM 41 trait groups were simulated

simultaneously considering H_I between 20 and 60 μmol photons m^{-2} s^{-1}. Depicted are the results at 0.75 m depth after 100 yr development time. The units of C_T and Tr_{av} are mgC m^{-3} and μmol photons m^{-2} s^{-1}, respectively.

Figure D: Results from a two species model

In addition to the community models RM and AM we employed a two-species model considering only two distinct phytoplankton species. The simulations use the same parameterization of phytoplankton as in RM in the main text including the trade-off between H_I and H_N (see also Fig. S3 blue line in S3 appendix). The condition of co-existence in the two-species model is investigated based on the ratio R_{2spec} of the vertically integrated concentrations C_{spec1} and C_{spec2} of species 1 and 2, respectively:

$$R_{2spec} = \frac{\min\left(\int C_{spec1}dz, \int C_{spec2}dz\right)}{\max\left(\int C_{spec1}dz, \int C_{spec2}dz\right)}, \qquad \text{(S6.1)}$$

We use $R_{2spec} > 10^{-4}$ as indication of co-existence of the two species.

The consequences of trophic state, characterized by N_{ini}, for co-existence in a system consisting of only two competing phytoplankton species with fixed traits were explored using the two-species model. Vertical turbulent diffusivity was assumed to be $K_z = 5\ 10^{-5}$ m^2 s^{-1}. The simulation results reveal that the abundance ratio R_{2spec} exceeds 10^{-4} for several combinations of master trait values H_I (Fig. S6) suggesting co-existence of the two species in all these cases. At same trophic state, i.e. same N_{ini}, the two species co-exist for several combinations of H_I (Fig. S6). Furthermore, co-existence occurs for a wide range of trophic states, e.g. at N_{ini} from 10 to 70 mgPm^{-3} (Fig. S6C). In contrast, competition in the multi-species community model leads for each N_{ini} to the selection of a unique single trait group or a unique combination of two traits groups in case of evolutionary stable co-existence (Fig. 3). At the same mixing conditions as in the two-species model ($K_z = 5\ 10^{-5}$ m^2 s^{-1}) evolutionary stable co-existence only occurs if $N_{ini} < 50$ mgP m^{-3} (Fig. 2 and 3).

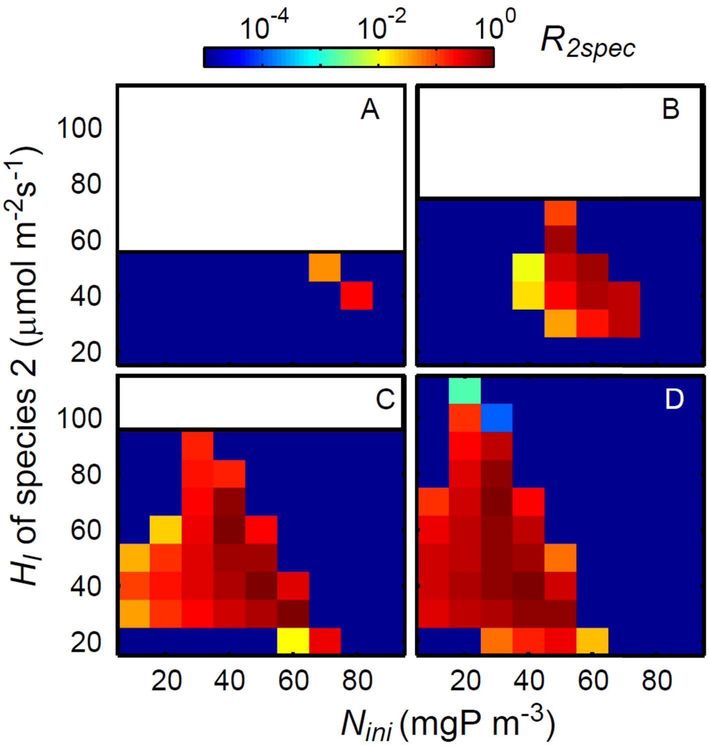

Figure D: The conditions for co-existence of two competing species obtained from the two-species model. Depicted are R_{2spec} for different combinations of two traits after 100 yr development time. In panels A to D, H_I of species 1 is 60, 80, 100, and 120 μmol photons m^{-2} s^{-1}, respectively. In all simulations turbulent diffusion was $K_z = 5 \cdot 10^{-5}$ m^2 s^{-1}.

The trophic state (N_{ini}) under which two competing species can co-exists depends on the specific values of the master trait chosen for the two species (Fig. S6). Co-existence can occur at much higher N_{ini} than possible for evolutionary stable co-existence of two species developing from selection within the phytoplankton community. Thus, the common approach to investigate competition of two species (e.g. Yoshiyama et al. 2009; Ryabov and Blasius 2014) cannot be employed to investigate environmental conditions under which evolutionary stable co-existence is possible. Furthermore, our results suggest that the selection of species in trait-based models not

only depends on the trade-off functions between trades but also on the eco-physiological limits of the trait space for the community present.
